# Genetic tests of ancient asexuality in Root Knot Nematodes reveal recent hybrid origins

**DOI:** 10.1186/1471-2148-8-194

**Published:** 2008-07-07

**Authors:** David H Lunt

**Affiliations:** 1 Department of Biological Sciences, University of Hull, Hull, HU6 7RX, UK

## Abstract

**Background:**

The existence of "ancient asexuals", taxa that have persisted for long periods of evolutionary history without sexual recombination, is both controversial and important for our understanding of the evolution and maintenance of sexual reproduction. A lack of sex has consequences not only for the ecology of the asexual organism but also for its genome. Several genetic signatures are predicted from long-term asexual (apomictic) reproduction including (i) large "allelic" sequence divergence (ii) lack of phylogenetic clustering of "alleles" within morphological species and (iii) decay and loss of genes specific to meiosis and sexual reproduction. These genetic signatures can be hard to assess since it is difficult to demonstrate the allelic nature of very divergent sequences, divergence levels may be complicated by processes such as inter-specific hybridization, and genes may have secondary roles unrelated to sexual reproduction. Apomictic species of *Meloidogyne *root knot nematodes have been suggested previously to be ancient asexuals. Their relatives reproduce by meiotic parthenogenesis or facultative sexuality, which in combination with the abundance of nematode genomic sequence data, makes them a powerful system in which to study the consequences of reproductive mode on genomic divergence.

**Results:**

Here, sequences from nuclear protein-coding genes are used to demonstrate that the first two predictions of ancient asexuality are found within the apomictic root knot nematodes. Alleles are more divergent in the apomictic taxa than in those species exhibiting recombination and do not group phylogenetically according to recognized species. In contrast some nuclear alleles, and mtDNA, are almost identical across species. Sequencing of Major Sperm Protein, a gamete-specific gene, from both meiotic and ameiotic species reveals no increase in evolutionary rate nor change in substitution pattern in the apomictic taxa, indicating that the locus has been maintained by selection.

**Conclusion:**

The data strongly suggests the tropical root knot nematode apomicts have a recent origin and are not anciently asexual. The results support that interspecific hybridization has been involved in the origin of this asexual group and has played a role in shaping the patterns of genetic diversity observed. This study suggests that genetic signatures of ancient asexuality must be taken with caution due to the confounding effect of interspecific hybridization, which has long been implicated in the origins of apomictic species.

## Background

The evolution of sexual reproduction and its maintenance in so many extant taxa has attracted much attention both as a major innovation of eukaryotes and a process that fundamentally shapes the diversity on which natural selection acts [1]. Sexual reproduction is so ubiquitous that some debate has focused on whether metazoan lineages can really exist for protracted evolutionary periods without it [2-6]. Yet despite this several groups have been suggested to persist without sexual reproduction for long periods [4]. The existence of asexual taxa over such timescales appears to present problems for the body of theoretical work concerning the evolution and maintenance of sexual reproduction [1].

### The genetic signatures of asexuality

**Figure 1:**
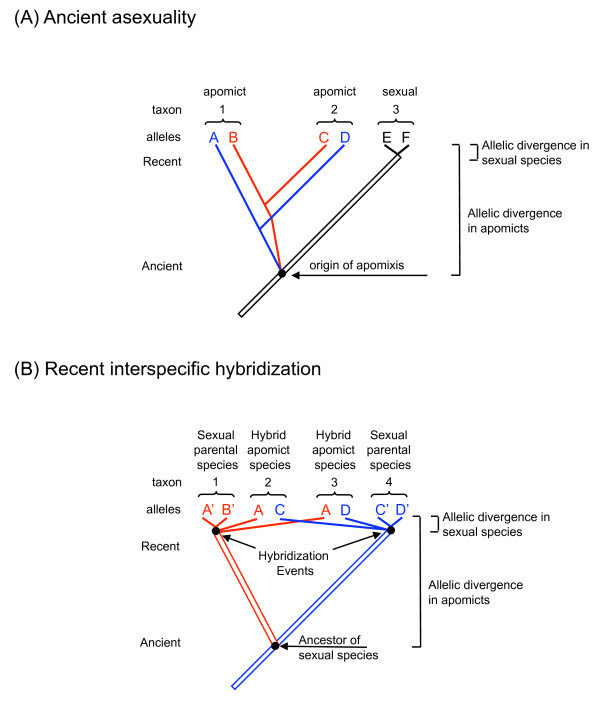
**Allele and species trees under differing modes of reproduction**. The diagram illustrates the similarity of (A) ancient asexuality (B) recent interspecific hybridization followed by apomictic reproduction in terms of "paralogous" segregation of alleles, and large allele sequence divergence (ASD) in asexual versus sexual species. Hatching denotes the action of recombination. Panel B shows a subset of possible diploid hybrid apomicts from two independent hybridization events. Triploid hybrids would show a mixture of large and small ASD within the same individual. The two hybrid apomicts shown possess some identical alleles between species, while exhibiting large ASD in the same individual. Parts of 1A redrawn from [7].

Asexual reproduction solely by mitotic division (apomixis), when compared to species that undergo meiotic recombination (sexuality), makes a number of predictions concerning the genetic diversity within these species. One such prediction is that of large allelic sequence divergence (ASD, "Meselson Effect") between "alleles" in apomictic lineages [7]. This happens because apomictic alleles do not recombine and coalesce in a reticulate manner as they would in sexual taxa, rather diverging by the accumulation of mutations (Figure 1).

A second consequence of asexual reproduction without recombination is that phylogenetic analysis of sequence data produces an allele tree that may not match the species tree accepted for these lineages. This "paralogous allele effect" (PAE) is observed because "alleles" in asexual species behave more like paralogous loci do in a sexual species [7]. Thus the closest relative of an allele's sequence in one species will be the orthologous allele in another species, rather than the other (paralogous) allele sequence within the same species. This observation of PAE can, along with ASD, be a strong signature of long-term asexual reproduction.

If apomixis arises in a diploid individual that is heterozygous (AB) at a locus, then without the action of recombination, "alleles" A and B are independent in their accumulation of mutations and their gene sequences will divergence from one another (Figure 1A). In effect, A and B behave as different paralogous loci do in sexual taxa. Sequence divergence will correspond to the time since the alleles last coalesced prior to the switch to apomixis. Since there is no transfer of gametes nor recombination between individuals, A and B are also independent of similar sequences in conspecifics, and have separate evolutionary trajectories. The divergence of A and B within a given individual (or apomictic species) is expected to be much greater than the divergence between alleles in a sexual species, where recombination operates and the coalescence of the alleles will be much more recent than the formation of the species. Apomictic individuals in a population are not related by the homogenizing processes of mating and recombination, but by descent from a single common ancestor with diversity due to the accumulation of mutations. Divergence between two copies of allele A, one from an individual in lineage 1 and one from an individual in lineage 2, will date to the divergence of those two lineages from a common ancestor. The divergences between alleles within an individual are expected to be greater than (predate) or equal to the divergences between lineages. This is similar to the concept of segregating ancestral polymorphism that can confound phylogenetic analysis at some loci of sexual species, although it is a characteristic of the entire genome of apomictic organisms and may persist over much greater timescales.

Ancient asexuality may not be the cause of large ASD if the origin of apomixis is interspecific hybridization (Figure 1B). In many parthenogenetic vertebrates [8], and other parthenogenetic taxa [9-14] interspecific hybridization has been implicated in the origins of asexuality and the asexuals may carry genes from two parental species. In such a case an apomictic lineage might possess alleles A, B, C, or D at a particular locus since the two parental species may not have shared alleles. In this situation, the pairs of alleles within each sexual parent (A and B; C and D) would share an ancestor within that species and not be highly diverged, whereas the divergence of the two sets of alleles (A, B *vs *C, D) would depend on the age of separation of the two parental species [15] and might be very large. Figure 1B illustrates the situation for diploid hybrid apomicts. Triploids would contain both alleles from one parent and thus have a mixture of closely related and highly divergent ASD values. Extreme ASD results from the cessation of recombination, either due to ancient asexuality or the independent evolution of diverged species that subsequently produce a hybrid. Since the causes and genetic signatures are very similar it is important to carefully distinguish between these alternatives, especially since the existence of ancient asexuality is so important and controversial for evolutionary biology [16].

An additional prediction of long-term asexual reproduction is that genes involved only in meiosis and associated processes (e.g. gamete production, sexual dimorphism) will have no required function and will not be actively maintained by selection [17]. In such genes mutations are expected to accumulate at an accelerated rate, the ratio of non-synonymous to synonymous substitutions (dN:dS) is expected to be different from that in genes under selection, and pseudogenisation (indels, frameshifts, stop codons) is likely to result. Although great progress is being made in identifying meiotic genes their roles in other processes are sometimes hard to exclude and different taxonomic groups may use the generally well-conserved meiotic machinery in different ways [18,19]. An alternative to meiosis-specific genes is the investigation of genes involved in gamete production. Nematodes possess amoeboid (crawling) sperm and the major sperm protein genes (*msp*) are a relatively well-investigated gene family with a major role in sperm structure and movement [20-22]. In *Caenorhabditis elegans *this sperm protein has also been shown to act as an extracellular signal for the completion of oocyte meiosis and contraction of the gonadal sheath cells, which is required for ovulation [23]. These two activities are determined by separate parts of the *msp *amino acid sequence [23]. Expression of *msp *is restricted to the sperm and spermatocytes in *C. elegans *and has been shown to be male specific in other species of nematodes [24-26]. Root knot nematode species are known to reproduce by both sexual and asexual means [27] and their *msp *may be a good candidate to examine the consequences of asexual reproduction for genes exclusively associated with sexual reproduction.

### Root Knot Nematodes

Root knot nematodes (RKN) of the genus *Meloidogyne *are very widely distributed plant pathogens that are estimated to be responsible for worldwide annual crop losses of ~5% [28]. The genus contains in excess of 80 species that differ in morphology, host-range, geographic distribution, cytology and mode of reproduction [29,30]. The three most agriculturally widespread and damaging taxa (*M. incognita, M. javanica and M. arenaria*) are obligatory apomicts in which bivalent chromosomes have never been detected [27] and cell division is exclusively mitotic [31]. Extensive aneuploidy is observed among populations of apomictic RKN [32] a state that has been predicted to be common in species that have lost meiosis [7]. In addition, the chromosomes of these apomicts cannot be placed into homologous pairs (as they can in automictic and sexual *Meloidogyne *species) and variation in chromosome size follows a continuous rather than discrete size pattern, indicating that many instances of karyotype change have occurred [32,33]. The decay of homologous chromosome pairs in ameiotic species has previously been identified as a key characteristic of truly asexual organisms [7,17,34] and such karyotype evolution may be rapid [35]. The apomictic RKN form "dyads" characteristic of mitotic divisions, and exhibit a single maturation division forming one polar nucleus and one egg nuclear each with the somatic chromosome number [27]. Cytological evidence therefore strongly supports their status as obligatory mitotic (apomictic) parthenogens that cannot go through meiosis.

The apomictic RKN, like many apomicts [36-39], produce occasional individuals that are morphologically male. Although these have sometimes been observed to introduce spermatozoa into females this resulted in the degradation of the sperm nucleus [32] and reproduction was still mitotic. The *Meloidogyne *apomicts have been highlighted as putative ancient asexuals [4], although their age has never been clear. Esbenshade and Triantaphyllou [40] make tentative estimates of age of divergence within *Meloidogyne *from multi-enzyme banding patterns. Using their figure 2 one can infer that the apomictic *Meloidogyne *may be approximately 17 million years old.

Perhaps surprisingly since many suggestions for the benefits of sex stress the importance of novel genotypic combinations in host-pathogen competitions, apomictic RKN are exceptionally wide ranging and successful pathogens. The potential host range for RKN may include most of the estimated 250,000 species of flowering plants, and a plant-nematode arms race of virulence and resistance is ongoing [41]. An additional apomictic species is *M. mayaguensis*, although this may be distinct from the three main species of apomictic RKN [42]. Also of economic concern, especially in temperate regions, are the facultative (automictic) meiotic parthenogens *M. hapla *(race A), *M. fallax *and *M. chitwoodi*. These species posses homologous chromosomes, go through a standard meiosis involving bivalents, and restore the somatic chromosome number (when reproducing by automixis) by fusing the second polar nucleus with the egg pronucleus [27,29,43].

Here *Meloidogyne *species with and without meiosis are compared to determine the effects of asexual reproduction on genomic diversity. Sequence analysis of three nuclear protein-coding genes is carried out to examine differences in ASD and PAE between the different reproductive modes. In addition a sperm-specific gene is sequenced and compared between the species utilizing sperm in their reproduction (facultative meiotic parthenogens) and those for whom sperm appear to have no function (obligatory mitotic parthenogens).

## Results

### Structure of loci

Elongation Factor 1-alpha primers amplified an approximately 780 bp fragment that included sequence from 4 exons and 3 introns. RNA polymerase II large subunit primers amplified an approximately 710 bp fragment that included coding sequence from 2 exons and a single intron. Dystrophin primers amplified a fragment of approximately 670–770 bp consisting of 3 exons and 2 introns. Major Sperm Protein primers amplified approximately 370 bp comprising 2 exons and a single intron. Intron boundaries conformed to the *C. elegans *consensus splice sequence [44]. The introns themselves contained indels and were considerably more A+T-rich than exons, a feature known to be characteristic of *C. elegans *introns. mtDNA primers amplified a 773 bp fragment of the *Meloidogyne *non-coding region between the 102 bp and 63 bp repetitive arrays, corresponding to bp 5419–6192 of Okimoto et al. [45].

Sequences have been deposited with the international databases with accession numbers EU699810–EU699942.

### Phylogenetic analysis

**Figure 2:**
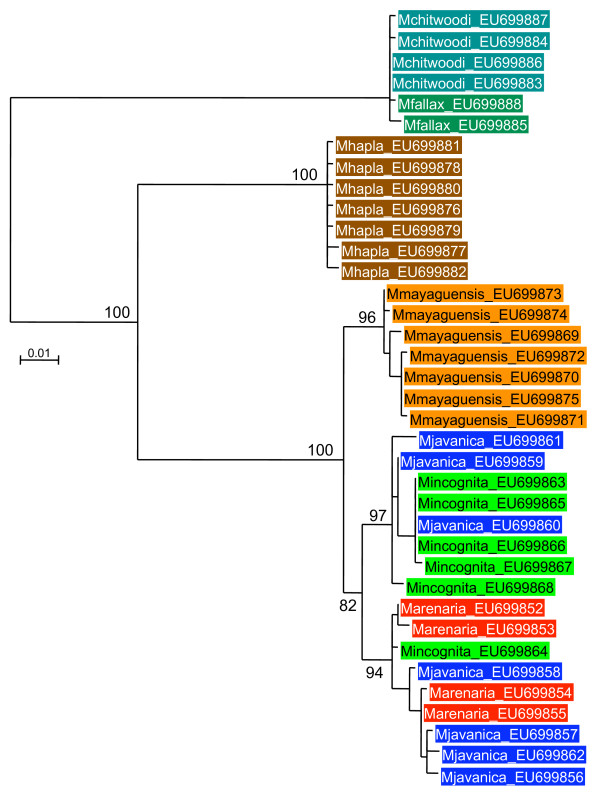
**Maximum likelihood phylogeny of RNA polymerase II exon sequences**. An alignment of 661 bp of exon sequence was analyzed with approximate likelihood ratio test support values given on key nodes.

**Figure 3:**
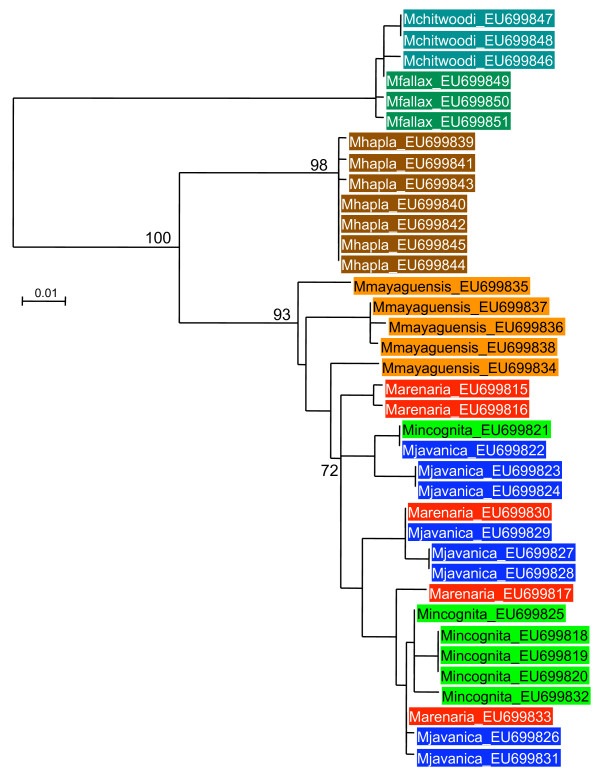
**Maximum likelihood phylogeny of dystrophin exon sequences**. An alignment of 549 bp of exon sequence was analyzed with approximate likelihood ratio test support values given on key nodes.

**Figure 4:**
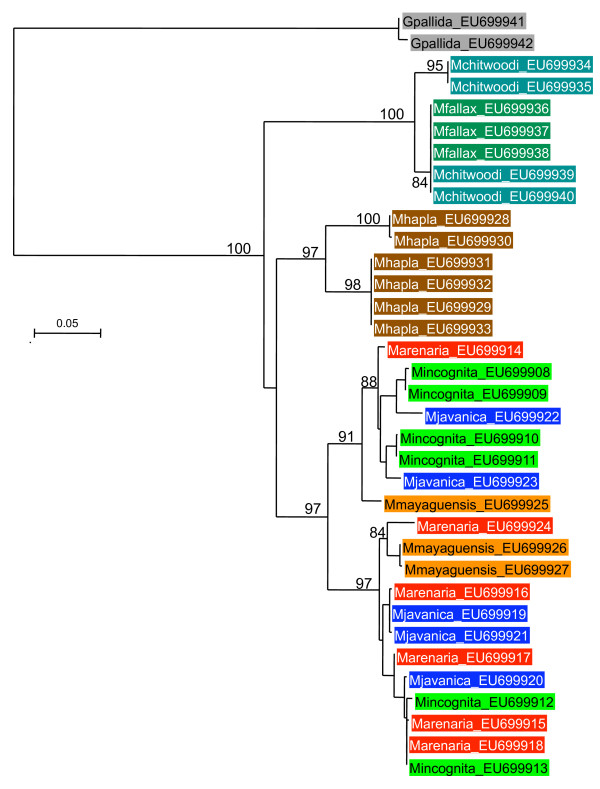
**Maximum likelihood phylogeny of elongation factor 1-alpha exon sequences**. An alignment of 639 bp of exon sequence was analyzed with approximate likelihood ratio test support values given on key nodes.

Alignments and phylogenetic analyses were carried out on exon sequence data to minimize the influence of alignment ambiguities in the indel-rich intron regions. The alignments of coding regions contained no indels and were unambiguous for all genes. Models of evolutionary change selected by MODELGENERATOR [46] using the AIC1 criterion were GTR+G (dystrophin), TVM+G (RNA polymerase II), TIM+G (elongation factor, *msp*). These were used in maximum likelihood phylogenetic reconstruction although gross tree topology was observed to be stable across a range of different models. Trees for all genes were similar in the phylogenetic relationship between groups recovered (Figures 2, 3, 4). There was generally good support for grouping the tropical apomictic species (*M. incognita*,*M. javanica *and *M. arenaria*) in a single clade, with the other apomict *M. mayaguensis *very closely related. In two of the three phylogenies *M. mayaguensis *was clearly distinct, although in the EF1-alpha tree its alleles were not resolved from those of the other three species. The apomictic species were placed as a sister group to the automict *M. hapla*. The (automictic) meiotic parthenogens *M. fallax *and *M. chitwoodi *were found to be very closely related and together formed the most basal grouping well diverged from the other automict *M. hapla*. Tree topologies were stable to the use of *Globodera pallida*, *Pratylenchus penetrans*, *C. elegans *as outgroup, or midpoint rooting.

### Paralogues of elongation factor 1-alpha

The EF1-alpha phylogeny (Figure 4) indicates that gene duplication might have occurred with some sequences representing paralogous loci. The alleles for both *M. hapla *and the *M. chitwoodi*-*fallax *complex each form two clusters with divergences greater than observed for other loci. An Ensembl blastp search with the translation of a *M. incognita *sequence revealed two matches in the *C. elegans *genome, one on chromosome III and the other on chromosome X, indicating that duplicate EF1-alpha genes have been evolutionarily conserved in nematodes. The differences between paralogues in all species are small at the amino acid level and the clustering of EF1-alpha sequences within species indicates that concerted evolution must have played a role in homogenizing these paralogous copies. If this were not the case then one would see a very basal dichotomy with the species grouping reflected within each of those two groups.

### Diversity levels

Phylogenetic analysis (Figures 2, 3, 4), and Table 1, indicates that within-species divergence was relatively low for automictic species. The exception to this was for elongation factor 1-alpha. Here diversity was similar within all species, although, as discussed above, it is likely that duplicate (paralogous) gene copies have been sequenced (Figure 4). Within each phylogenetic gene copy of EF1-alpha diversity was again low for automict species. More diversity was present between sequences in the apomict group. This diversity was not partitioned between species, with identical exon sequences being shared between different species for both RNA polymerase II and dystrophin (Table 1).

**Table 1:** Intraspecific diversity of dystrophin and RNA polymerase II genes.

	N	Minimum number substitutions	Maximum number substitutions	Mean number substitutions	Number substitutions to closest relative
RNA polymerase II					

***M. incognita***	4	1	15	8.2	0 *M. javanica*
***M. javanica***	7	3	16	10.5	0 *M. incognita*
***M. arenaria***	4	1	9	5.5	2 *M. javanica *2 *M. incognita*
***M. mayaguensis***	6	1	5	3.2	16 *M. javanica*
*M. hapla*	7	2	4	2.3	60 *M. mayaguensis*
*M. fallax*	2	3	3	3	1 *M. chitwoodi*
*M. chitwoodi*	3	1	2	1.8	1 *M. fallax*

Dystrophin					

***M. incognita***	4	5	31	15.3	3 *M. arenaria*
***M. javanica***	6	2	30	21.4	0 *M. arenaria*
***M. arenaria***	5	2	32	20.9	0 *M. javanica*
***M. mayaguensis***	5	1	36	22.7	24 *M. arenaria*
*M. hapla*	6	1	2	1.7	64 *M. incognita*
*M. fallax*	3	3	4	3.3	2 *M. chitwoodi*
*M. chitwoodi*	2	5	5	5	2 *M. fallax*

Divergences are measured as absolute number of uncorrected differences. Apomictic species are in bold. N represents the number of sequences and only 1 comparison is available for *M. fallax *RNA polymerase II and *M. chitwoodi *dystrophin.

Five mtDNA sequences representing the three tropical apomictic RKN were obtained and added to the published *M. javanica *sequence [45] making an alignment of 614 bp. The mitochondrial sequences were extremely AT-rich (~80%) and sequence divergences were low (<2%) between the isolates sequenced and by comparison to a published sequence [45]. No amplification was obtained with these primers in any automict. The region of the mitochondrial genome sequenced does not have a recognizably homologous counterpart in *M. hapla *[47] and presumably not either in the even more divergent *M. fallax *and *M. chitwoodi*.

### Diversity and relative rates of *msp *genes

**Figure 5:**
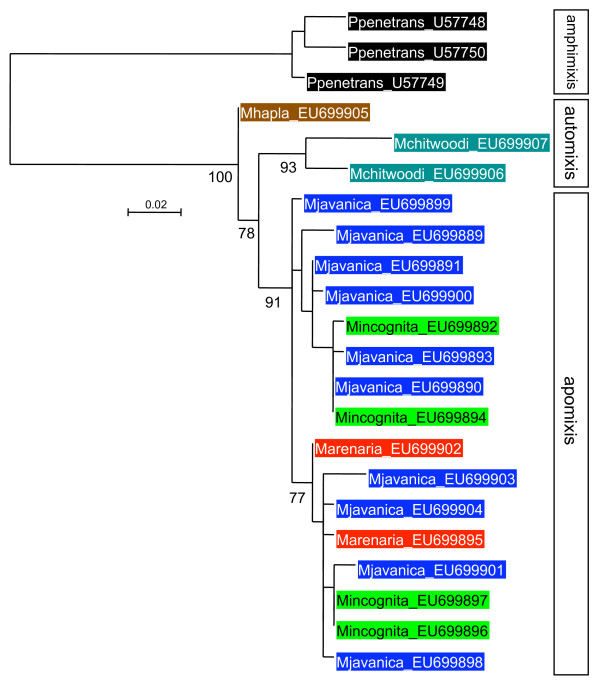
**Maximum likelihood phylogeny of major sperm protein exon sequences**. An alignment of 256 bp of exon sequence was analyzed with approximate likelihood ratio test support values given on key nodes.

**Figure 6:**
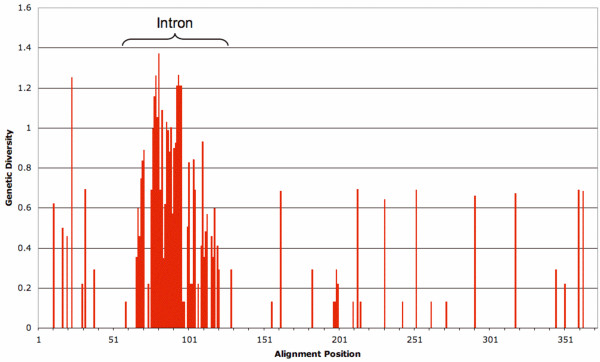
**Genetic diversity**. Shannon's alignment uncertainty or "entropy" [67] is plotted along an alignment of apomictic *msp *sequences illustrating the variability of the intron with respect to exons.

The *Meloidogyne *major sperm protein exon dataset (Figure 5) was examined for relative rates of substitution for all pairwise combinations of taxa with respect to the outgroup (*Pratylenchus penetrans *MSP3). No significant test was observed, with p-values for the apomictic sequences ranging from 0.147–1.000, indicating that there is no increased rate of evolution in apomictic *msp *genes. Fifteen substitutions in apomictic *msp *genes were synonymous and a further seven were non-synonymous. Alignment of intron sequences, even within the apomict group, was not straightforward, with small changes in gap weight penalties or manual alignment choices influencing the location of indels and diversity statistics recovered. It was notable that the 5' and 3' ends of the introns aligned almost unambiguously and that this alignment confidence decayed rapidly once outside of the regions usually associated with the intron splicing sequences. Although exact quantification of genetic distances without subjective alignment decisions was difficult, intron sequences were clearly much more divergent than exon sequences for all automated or manual alignments considered. A plot of sequence diversity along *msp *(Figure 6) highlights the relative diversity of the intron.

Finally, in order to compare the pattern of sequence change, the *msp *sequences were divided into two groups (a) apomicts (b) automicts plus outgroup (*P. penetrans*) sequences. The hypothesis that there was no difference in the pattern of substitutions (dN, dS) between these two groups was tested in a codon-based maximum likelihood framework. This hypothesis could not be rejected by a likelihood ratio test (p = 0.718).

## Discussion

It is clear that apomictic *Meloidogyne *contain divergent alleles in comparison to the meiotically recombining taxa, with ASD values larger than those found in automicts (Table 1). In addition the phylogenetic signal of alleles within the apomict taxa does not recover species designations identified by traditional methods. These observations are characteristic of the predictions of ASD originally put forward by Meselson and developed by Birky [7] and Judson and Normark [4]. In contrast to the expectations of ancient asexuality however, no evidence is found of 'decay' of the gamete-specific *msp *sequences. How might this apparent contradiction be explained?

Causes other than very prolonged periods of asexuality may also result in the observed difference in ASD between apomictic and automictic species. Two of these with relevance to the *Meloidogyne *system are the reduction of diversity in automict species and interspecific hybridization.

### Diversity levels in automictic species

Recent work examining the genetics of *M. hapla *[48] has highlighted the potential for the automictic meiotic parthenogens to rapidly lose diversity. In this mode of reproduction *M. hapla *regains diploidy by fusing terminal products of meiosis that contain sister chromatids. Thus the diploid genome will be homozygous except in those locations in which both recombination has occurred and where the chromosomes were initially different at the start of the first meiotic division. Where apomictic taxa are closely related to automictic relatives, including the *Meloidogyne *system presented here, allelic divergences may be exaggerated in apomictic species by comparison to automictic species for which the breeding system keeps allelic divergences low or absent.

### Interspecific hybridization

An alternative explanation for large ASD values in apomictic species is interspecific hybridization. It is thought that many apomictic species may be of hybrid origin, a process that can prevent normal meiosis [49,50]. The three most agriculturally important RKN apomicts, *M. incognita*, *M. arenaria *and *M. javanica*, have been suggested before to have arisen as a result of hybridization between unspecified automict or sexual taxa [43,51,52]. Certainly, if these taxa possess hybrid genomes their parental species have not been sampled in this study since their alleles do not cluster with those from any other species. This may not be too surprising however since although there are more than eighty species of *Meloidogyne *described, only a small number of the major agricultural pathogens were surveyed here.

Evidence of possible interspecific hybrid origins for apomictic RKN comes from analysis of nuclear rDNA and internal transcribed spacer sequences [53]. This reveals a pattern of diversity concordant with those presented here from nuclear protein coding genes. Apomictic species show both very divergent ribosomal ITS sequences within individuals and very similar ones between species. Ribosomal ITS sequences from apomictic species can be placed into two deeply diverging groups, which presumably represent the divergence of the parental species.

If the major sperm protein has no novel function in apomictic RKN then the pattern of diversity observed across the *msp *sequence alignment (Figure 6) provides strong evidence for interspecific hybridization. Since this is data from exclusively apomictic species with a monophyletic origin the mutations observed should have accumulated since the common origin of those taxa. Without a requirement for functional major sperm protein these mutations should be approximately randomly distributed, irrespective of whether the origin of the taxa was recent or ancient. The substitutions however clearly cluster within the intron, strongly suggesting that they have accumulated over a period in which purifying selection was operational. If these *msp *alleles had initially diverged in sexual species, and had subsequently been brought together in an interspecifc hybridization event, one would predict exactly the pattern observed in Figure 6 where the majority of substitutions occur in the intron.

Although there can be large sequence divergences between gene copies in a single apomictic RKN individual there is also striking sequence similarity between apomictic species at some, but not all sequences. Genes may show larger sequence diversity between "alleles" within individuals than between the same sequences in different species (Table 1). Thus *M. arenaria *has a dystrophin sequence also shared by *M. javanica*, although other *M. arenaria *sequences differ by as many as 32 substitutions from the first. For RNA polymerase *M. javanica *and *M. incognita *also posses some identical sequences, although alleles within these species may differ by 16 changes. If these asexual species were truly ancient, then one would expect the minimum sequence divergence to be much greater, reflecting the many mutations since they last shared a common ancestor. Similarly, mitochondrial DNA studies based on RFLPs have indicated very low levels of sequence divergence between these species [54], and sequencing of 614 bp of non-coding mtDNA in this study also revealed the three tropical apomicts to be very similar.

The ASD data presented here is compatible with the predictions of interspecific hybdridization events, an idea also discussed by Hugall et al [53] and Castagnone-Sereno [51]. If females from an ancestor of the tropical apomicts engaged in interspecific hybridization with males from a distinct species then sibling lineages would emerge and might today represent the different apomictic species. They ought to show several genetic signatures of these hybridizations. First, they should initially share very similar mtDNA. Second, the nuclear loci within individuals would exhibit instances of high ASD, reflecting the independent divergence of alleles in two stages; (a) divergence in different species up until the hybridization event and (b) divergence since that event due to the absence of recombination. Thirdly, nuclear loci within individuals would exhibit instances of low ASD, reflecting that they were, until the hybridization event, recombining alleles in a sexual parental species. Fourthly, alleles should exhibit PAE and not cluster by species since these are mosaic genomes and individuals have not experienced the type of coalescence that we observe in sexual species. All these predictions have been met.

If multiple hybridization events account for the origins of the apomictic species this can occur in two ways. It is very likely that since the apomicts share closely related mtDNA types that their maternal parent was the same or very closely related. The first option is that females from this parent species mated with males from several other distinct species. The alternate situation is that females from the parent species mate with males from a single other species. These two hypotheses make different predictions. One would expect that alleles from the female parent would be found in all apomictic taxa and form a multi-species clade on the tree. In the first case, alleles from the distinct male parental species would form multiple discrete clades on the tree each containing only individuals from the single apomictic species descended from that hybridization event. This is not observed in this study, nor that of Hugall et al. with rDNA sequences [53]. Unless we say that the parental species were very closely related and their alleles were not phylogenetically distinct even before the hybridization this multi-species origin for the apomicts can be rejected. The second case predicts that no single-species clades will be found, as is observed in this study. The tools for identifying the parental species are now clear. They will be sexual, or facultatively so, which can be detected by the presence of homologous chromosome pairs. Both species will have nuclear alleles that cluster within the diversity found at the four nuclear loci of the apomictic species. The female parent will share mtDNA with the apomicts while the male parent will have distinct mtDNA.

### Major Sperm Protein genes in asexual *Meloidogyne*

It might be anticipated that if meiosis had been abandoned in *M. arenaria*, *M. javanica *and *M. incognita *their major sperm protein genes would be redundant, and hence under relaxed selection. It would follow then that there would be no particular penalty for non-synonymous changes, frameshifts, or stop codons, and they would evolve much faster than genes under strong selection to maintain function (*msp *in sexual species). In contrast to this prediction the *msp *genes in apomicts showed none of these types of change. Although pseudogenes are common in large multigene families such as *msp *[22], the *msp *genes sequenced from apomictic RKN are unlikely to be pseudogenes, with no frameshifts or stop codons observed. As part of this study DNA database searches of previously published EST collections [55] were carried out and indicate that *msp *genes are still expressed in apomictic *M. incognita*. Relative rate tests indicate that the sequences from apomictic taxa are evolving at a similar rate to those in sexual species, and analysis of the substitution patterns in *msp *sequences from apomictic and meiotic species cannot reject that they are the same. Finally, most substitutions since the coalescent of the apomict sequences occur in the intron, as is typically observed for sequences under purifying selection. The intron does not appear to be homogeneous however with relatively few indels or substitutions occurring at the intron boundaries in the sequence regions typically identified as being important for correct intron splicing. These patterns of intron diversity were also observed in the three other nuclear genes sequenced indicating again that they are typical of actively maintained genes. The primary function of major sperm protein genes is in the formation of sperm, their expression being restricted to sperm and spermatocytes [20,25]. These sperm proteins are known to act as extracellular signals in *C. elegans *and are involved in both the resumption of oocyte meiosis (maturation) and sheath cell contractions involved in ovulation. These two functions are known to be dependent on different regions of the peptide sequence with amino acids 1–106 influencing maturation but not ovulation and amino acids 106–126 only ovulation [23]. Since the *msp *sequences obtained in this study correspond to amino acids 24–106 the functions of this regions are likely to be restricted to those involved in sexual reproduction (sperm structure/movement and the completion of the oocyte meiosis). Although it could be that this section of *msp *has an additional role unrelated to sexual reproduction, which might still be essential in ameiotic species, there is no evidence of this in *C. elegans *or any other nematode. In addition, a rapid change of function (such as the loss of the two major functions of *msp *with the transition to apomictic reproduction) might be expected to be accompanied by a rapid change in evolutionary rate or dN:dS with respect to close sexual relatives, neither of which is observed. Together these results provide compelling evidence that apomictic RKN *msp *sequences are either maintained by selection or that they have only very recently escaped from such control. Change in gene function might not necessarily require large changes in evolutionary rate or amino acid sequence however, with more subtle changes in sequence or regulation also able to bring about functional change. Detailed study of the expression and exact role of major sperm proteins in meiotic and ameiotic species will be required to properly elucidate its action. In summary then, although the data from ASD is compatible with either ancient asexuality or recent hybrid origins, the lack of synonymous site sequence divergence, low mtDNA divergence and *msp *sequence data are only compatible with a recent origin of asexuality.

### How ancient are the apomictic RKN?

The evidence presented here indicates that although some of the genetic signatures of long-term asexuality appear to be fulfilled for RKN, they are more likely to be recent asexuals. No degeneration of *msp *sequences in apomictic taxa is observed; with dS>dN and the clustering of mutations in the intron instead indicating that purifying selection has been the dominant force shaping sequence diversity. It is likely therefore that loss of a requirement for apomictic *msp *occurred very recently (or that the sequences have alternative functions strongly maintained by selection). From the almost identical alleles at nuclear loci and the very shallow mtDNA divergence levels we can reasonably conclude that these species are only recently diverged from one another. Although cryptic sexuality might currently seem unlikely for the tropical root knot nematodes, since careful cytological studies have revealed no evidence for it, several species previously thought to exhibit obligatory asexuality have been discovered to engage in some form of sexual reproduction [56-59]. Population genetic data from wild-caught individuals, or females with associated egg-masses, ought to be an excellent source of data to understand the nature of reproduction actually occurring in these and other *Meloidogyne *species.

If the genomes of these apomicts are hybrids between divergent species then assessing the coalescent point for diversity between apomicts using pooled multilocus data will give an age of separation of the parental taxa, not of the asexual species themselves. Consequently there will be a large overestimate of the age of origin of asexual lineages. Sequence based approaches, such as presented here, can treat sequences as separate entities and thus tease apart deeper and more recent divergences in the evolutionary history of this important group. It is clear from this work that the origin of the three main species of apomictic RKN is very recent indeed, since some sequences are shared between them without change, even at synonymous sites. Determining how recent this split is will require much more sampling, more loci, the development of well-calibrated molecular clocks and appropriate coalescent population genetics.

## Conclusion

The genetic signatures of ancient asexuality are often similar to those produced by interspecific hybridization followed by asexual reproduction, even if this has occurred recently. Since hybridization as a cause of apomixis is suggested to be common, extreme care is needed when examining putative ancient asexuals by genetic means. The apomictic tropical root knot nematodes despite sometimes-large allelic sequence divergence and a lack of clustering of alleles as predicted for ancient asexuals, are almost certainly recent asexuals. In support of this the apomict species show instances of very small ASD, with some alleles unchanged between different species, and the major sperm proteins, a gamete-specific gene family, show no sign of increased substitution rate, changed substitution pattern, nor pseudogene formation despite no apparent functional requirement for the maintenance of these sequences by natural selection. Taken together these results indicate that these species likely arose recently through interspecific hybridization events. The diversity of reproductive strategies present in the genus *Meloidogyne*, their rapid reproductive rate, ability to be cultured, rapidly increasing genomic resources and recent origins will make them a powerful system for the experimental study of the evolution and maintenance of reproductive modes.

## Methods

### DNA and amplification

DNA samples of isofemale nematode isolates were provided by colleagues V. Blok and M. Phillips (*M. incognita*, Martinique, USA; *M. javanica*, Spain, Portugal; *M. arenaria*, Ivory Coast, West Indies; *M. mayaguensis*, Puerto Rico; *M. hapla*, Netherlands; *M. fallax*, Netherlands; *M. chitwoodi*, Netherlands; *Globodera pallida*, UK), P. Roberts (*M. incognita*, USA), B. Hyman (*M. javanica*, USA). The *M. hapla *isolate was race A, reproducing by facultative meiotic parthenogenesis.

PCR primers (Table 2) were designed by the author to conserved regions of Elongation factor 1-alpha, RNA polymerase II large subunit, Dystrophin and a non-coding region of mitochondrial DNA (mtDNA), after alignment of sequences from the databases. The Major Sperm Protein gene (*msp*) was amplified using previously described primers [60]. These *msp *primers amplify amino acids 24–106 which comprises 77% of the region identified as responsible for acting as a signal for the resumption of meiosis, and none of the 20 amino acid region involved in signaling for sheath cell contraction [23].

**Table 2:** PCR Primers used in this study.

Gene	Primer	Sequence
EF1-alpha	EFA	5'-AAYATGTCNTGGTTYAARGGGTGG-3'
	EFB	5'-CCGACAGTNACNGTYTGGCKCATRTC-3'
RNA polymerase II	RP3F	5'-GAAGCTGTTATTGTTTCGGGAGAAG-3'
	RP3R	5'-AAGTTTATCTGCATTGTCATCTGTG-3'
Dystrophin	Dys1F	5'-AAAGAACAAAGGCTTTCAAGTATGT-3'
	Dys1R	5'-ACGCCGTGTTGGTCAACAAATAAAT-3'
mtDNA	Uni5P	5'-TATTTTTTAATAGTTAGTGTTGGTA-3'
	Uni3P	5'-TATATGATAAACTAAAACCGATCTT-3'
*msp *[60]	mspF	5'-GAAGATYGTCTTYAAYGCNCC-3'
	mspR	5'-GGATTCCATCWCCYTGGAACCA-3'

Major sperm protein primers originate from [60].

PCR reactions for all loci were typically carried out in a 10 μl volume with 1× PCR buffer, 1.5 mM MgCl_2_, 200 uM dNTP, 100 nM each primer and 2 units *Taq *polymerase. Cycling conditions were 94°C 3 mins; (94°C 45 s, 50°C 1 min, 72°C 2 mins) ×35; 72°C 10 mins.

PCR products were purified using Wizard spin columns (Promega) and 5 μl ligated into pGEM T-easy (Promega) or pCR2.1 (Invitrogen) cloning vectors. After transformation, the presence of inserts was confirmed by PCR using M13 primers. Plasmid DNA was prepared using QIAspin purification columns (Quiagen). Approximately 1 μg of plasmid DNA, or 1 μl of colony-PCR product, was used to inoculate a cycle-sequencing reaction using the ThermoSequenase kit (Amersham/Pharmacia). Template was sequenced on both strands using fluorescently labeled M13 primers.

### Alignment and phylogenetic analyses

Sequences were aligned using ClustalX [61] and intron boundaries identified by reference to the nematode splice signals described in Blumenthal and Steward [44] and the amino acid sequence of *C. elegans*. For the *msp *gene published sequences from close relative *Pratylenchus penetrans *was also included as outgroup [60]. Maximum likelihood phylogenetic analyses were carried out using PHYML v2.4.4 [62] using the optimal model of sequence evolution identified with MODELGENERATOR v0.83 [46] and approximate likelihood ratio test support values [63]. The maximum likelihood model was set with 4 substitution rate classes with gamma distribution parameter estimated by PHYML. The potato cyst nematode *Globodera pallida *(a close relative of *Meloidogyne*) was chosen as outgroup for the elongation factor 1-alpha tree whereas midpoint rooting was employed for RNA polymerase II and dystrophin trees. Using the described rooting procedures, midpoint rooting, or rooting with *C. elegans *made no change to the branching order of the major groups (unpublished results). Estimates of nucleotide diversity were obtained using DNAsp v4 [64]. Relative rate tests and comparison of codon based ML substitution processes for *msp *data were carried out with HYPHY v0.99 [65]. Relative rate tests were carried out using *P. penetrans *as the outgroup for all pairwise combinations of *Meloidogyne msp *sequences using the "PaiwiseRelativeRate.bf" test in HYPHY with codon-based substitution models. For the comparison of evolutionary change the species were divided into two pools; those exhibiting meiosis and those reproducing apomictically. The "TestBranchDNDS.bf" analysis in HYPHY was then employed to determine if the apomictic species evolved under different dN (nonsynonymous substitutions per nonsynonymous site) and dS (synonymous substitutions per synonymous site) than the rest of the tree. Absolute number of differences between exon sequences (Table 1) was calculated using PAUP* [66] with the data corrected to remove identical sequences recovered from the same isolate as these may represent multiple instances of the same sequence.

## Authors' contributions

DHL carried out all parts of this work
